# Functional Analysis and Marker Development of *TaCRT-D* Gene in Common Wheat (*Triticum aestivum* L.)

**DOI:** 10.3389/fpls.2017.01557

**Published:** 2017-09-11

**Authors:** Jiping Wang, Runzhi Li, Xinguo Mao, Ruilian Jing

**Affiliations:** ^1^College of Agronomy, Shanxi Agricultural University Jinzhong, China; ^2^National Key Facility for Crop Gene Resources and Genetic Improvement, Institute of Crop Science, Chinese Academy of Agricultural Sciences Beijing, China

**Keywords:** *Triticum aestivum* L., *TaCRT-D*, functional analysis, functional marker, single nucleotide polymorphism (SNP)

## Abstract

Calreticulin (CRT), an endoplasmic reticulum (ER)-localized Ca^2+^-binding/buffering protein, is highly conserved and extensively expressed in animal and plant cells. To understand the function of CRTs in wheat (*Triticum aestivum* L.), particularly their roles in stress tolerance, we cloned the full-length genomic sequence of the *TaCRT-D* isoform from D genome of common hexaploid wheat, and characterized its function by transgenic *Arabidopsis* system. *TaCRT-D* exhibited different expression patterns in wheat seedling under different abiotic stresses. Transgenic *Arabidopsis* plants overexpressing ORF of *TaCRT-D* displayed more tolerance to drought, cold, salt, mannitol, and other abiotic stresses at both seed germination and seedling stages, compared with the wild-type controls. Furthermore, DNA polymorphism analysis and gene mapping were employed to develop the functional markers of this gene for marker-assistant selection in wheat breeding program. One SNP, S440 (T→C) was detected at the *TaCRT-D* locus by genotyping a wheat recombinant inbred line (RIL) population (114 lines) developed from Opata 85 × W7984. The *TaCRT-D* was then fine mapped between markers *Xgwm645* and *Xgwm664* on chromosome 3DL, corresponding to genetic distances of 3.5 and 4.4 cM, respectively, using the RIL population and Chinese Spring nulli-tetrasomic lines. Finally, the genome-specific and allele-specific markers were developed for the *TaCRT-D* gene. These findings indicate that *TaCRT-D* function importantly in plant stress responses, providing a gene target for genetic engineering to increase plant stress tolerance and the functional markers of *TaCRT-D* for marker-assistant selection in wheat breeding.

## Introduction

Calreticulin (CRT) is an abundant Ca^2+^-binding protein predominantly localized in the endoplasmic reticulum (ER). CRT functional domain is highly conserved. A typical CRT protein contains three distinct structural and functional domains: a highly conserved globular N-domain which includes a cleavable signal peptide sequence; a P-domain which is responsible for high affinity and low capacity Ca^2+^-binding ability; and a C-domain which includes a (K/H)DEL ER retrieval signal (Michalak et al., 1999, 2009) and exhibits low affinity and high capacity Ca^2+^-binding ability.

The CRTs protein is ubiquitously expressed in all multi-cellular eukaryotes investigated (Michalak et al., 1999; Coe and Michalak, 2009), including humans, nematodes, fruit flies and plants (Jia et al., 2009). CRTs are mainly present in the ER (Opas et al., 1996), but also in the nucleus (Brünagel et al., 2003), the nuclear envelope (Napier et al., 1995), the cytosol (Afshar et al., 2005), the spindle apparatus of dividing cells (Denecke et al., 1995), the cell surface (Gardai et al., 2005), mitochondria (Shan et al., 2014) and plasmodesmata (Laporte et al., 2003; Chen et al., 2005), which indicates that CRTs take part in multiple cellular functions. In animals, extensive studies of CRTs have elucidated many key physiological functions inside and outside the ER (Michalak et al., 1999, 2009; Gold et al., 2010; Wang J.P. et al., 2012; Wang W.A. et al., 2012), such as intracellular Ca^2+^ storage, the regulation of ER Ca^2+^ homeostasis (Mery et al., 1996; Mesaeli et al., 1999; Persson et al., 2001), involvement in Ca^2+^-dependent signal pathways (Nakamura et al., 2001; Arnaudeau et al., 2002; Gelebart et al., 2005), molecular chaperone activity in the ER (Nigam et al., 1994; Nauseef et al., 1995; Jin et al., 2009; Jiang et al., 2014), control of cell adhesion (Opas et al., 1996; Johnson et al., 2001), angiogenesis (Ding et al., 2014), functions related to the immune system and apoptosis (Waterhouse and Pinkoski, 2007) as well as roles in pathogenesis (Qiu and Michalak, 2009).

In plants, CRT genes were initially identified from spinach leaves (Menegazzi et al., 1993). Subsequently, CRT cDNA clones were isolated from numerous plants including barley (Chen et al., 1994), tobacco (Denecke et al., 1995), maize (Kwiatkowski et al., 1995; Dresselhaus et al., 1996), Chinese cabbage (Lim et al., 1996), *Arabidopsis* (Nelson et al., 1997), castor bean (Coughlan et al., 1997), and rice (Li and Komatsu, 2000). Calreticulins are expressed in all meristematic and mature cell types, such as roots, young leaves, developing and germinating seeds, pollen, and other flower organs (Denecke et al., 1995; Nelson et al., 1997; Nardi et al., 2006; Suwińska et al., 2017), indicating that CRTs are ubiquitously involved in plant development regulations. Increasing data has evidenced that endogenous basal expression levels of both CRT mRNA and protein are upregulated in response to a wide range of stimuli, such as gravistimulation, cold, salt, drought, exogenous abscisic acid (ABA) treatment, and water stress (Heilmann et al., 2001; Persson et al., 2003; Komatsu et al., 2007; Jia et al., 2008; Kim et al., 2013). For example, the rice gene *OsCRO1* from CRT family was induced by low temperature as a response mechanism to cold stress (Li et al., 2003). These results demonstrate that CRT also functions in plant defense and stress responses.

Wheat, one of main cereal crops in the world, contains a huge and very complex genome consisting of three sub-genomes A, B and D. Drought and other environmental stresses greatly limit wheat (*Triticum aestivum* L.) growth and productivity worldwide. Understanding the molecular mechanism of stress response is highly required for wheat genetic improvement to increase stress tolerance and grain yields. As a conserved protein involved in Ca^2+^ signaling and protein folding, CRT may also play roles in wheat stress tolerance. Previously, our group isolated a full-length cDNA of *TaCRT* gene encoding a wheat TaCRT3 isoform (EF452301) from the ‘Hanxuan 10’ variety of hexaploid wheat, and heterologous overexpression of *TaCRT* in tobacco enhanced plant drought tolerance (Jia et al., 2008). Expression analysis indicated that *TaCRT1* (ADG85705), another wheat CRT isoform, may participate in wheat yellow rust resistance (An et al., 2011). Xiang et al. (2015) isolated three full-length cDNAs encoding wheat CRT1, CRT2, and CRT3 isoforms, namely *TaCRT1, TaCRT2*, and *TaCRT3-1*, respectively. *TaCRT1* (AY836753) overexpression could improve salinity tolerance in tobacco. Despite these reports showing wheat CRT involving in stress responses, it remains to address the details of wheat CRTs’ functions including multiple isoforms, distribution in genome, evolutionary redundancy, structure and function divergence, and how to use these *CRT* genes in wheat breeding program.

Therefore, the present study was conducted to investigate new function of TaCRT isoform (TaCRT-D) on D genome of wheat, particularly its roles in plant stress responses, by transgenic approach in *Arabidopsis* and expression analysis. Furthermore, DNA polymorphism analysis and gene mapping were employed to develop the functional genomic-specific and allele-specific markers of the *TaCRT* gene for marker-assistant selection in wheat breeding. DNA and cDNA clones of *TaCRT-D* were isolated from hexaploid wheat genome, and *TaCRT-D* expression was different in wheat seedlings under different stresses. Overexpression of *TaCRT-D* in *Arabidopsis* plants significantly increased plant stress tolerance. Moreover, SNPs were detected in the homeologous *TaCRT* loci on chromosomes 3A, 3B, and 3D. One of SNPs, S440 (T→C) was specific for the *TaCRT-D* locus which was fine mapped between markers *Xgwm645* and *Xgwm664* on chromosome 3DL using the RIL population and Chinese Spring nulli-tetrasomic lines. Finally, functional markers (FMs) for *TaCRT-D* isoform gene in wheat were developed. Our data provide novel information for understanding wheat CRTs’ functions and utilization of the excellent *TaCRT-D* isoform in wheat genetic improvement.

## Materials and Methods

### Plant Materials

Common hexaploid wheat (*Triticum aestivum* L.) genotype ‘Hanxuan 10’ with a higher drought-tolerant phenotype was used to study the expression of the target gene at different developmental stages under different stress treatments.

For *Arabidopsis* (*Arabidopsis thaliana* Columbia 3), the homozygous transgenic lines and the wild type lines were cultured in MS solid medium under 12 h light/dark cycle at 22°C. Separate samples were exposed to drought, salt, cold, and exogenous ABA stresses at different growth stages.

To identify the DNA polymorphisms of the *TaCRT* genes, a total of six accessions were used, including two common wheat cultivars (cv.) and four wild relative species. The two common cultivars are Opata 85 (*Triticum aestivum* L., AABBDD, 2*n* = 6× = 42) and W7984. The later is a synthetic wheat line derived from a cross of the durum cv. Altar 84 with an accession of *Ae. squarrosa* followed by chromosome doubling. The four wild relative species are *T. urartu* U203 (AA, 2*n* = 2× = 14), *Aegilops speltoides* Y2003 (BB, 2*n* = 2× = 14), *Aegilops tauschii* Y215 (DD, 2*n* = 2× = 14) and *T*. *polonicum* P09 (AABB, 2*n* = 4× = 28).

For chromosome location and genetic mapping of the *TaCRT-D* genes, a set of nulli-tetrasomic lines developed from Chinese Spring and a recombinant inbred line (RIL) population (Opata 85 × W 7984) were used. The 114 inbred lines used as the genetic mapping population were provided by the International Triticeae Mapping Initiative (ITMI). Leaf samples of all plant materials were harvested from 7-day-old seedlings and stored at -80°C.

### Gene Expression Analysis

After being sterilized with 75% ethanol and washed with sterilized water, the wheat seeds were germinated and cultured with water in a controlled-growth chamber (20°C, 12 h light/dark cycle). Seedlings at the two-leaf stage (9-day-old) were used for all subsequent treatments. For stress treatments, 250 mM NaCl was used for salt stress, 4°C for cold stress, and 50 μM ABA for exogenous ABA stress. The seedlings were stressed in different solution for 1, 3, 6, 12, 24, 48, and 72 h, respectively. The control seedlings were watered without treatment. Leaf samples were collected from the seedlings at different time points and frozen quickly with liquid nitrogen and stored at -70°C for RNA isolation and other analysis.

Total RNA was extracted using Trizol reagent and treated with RNase-free DNase. The real-time RT-PCR was performed in a 25 μL reaction mixture containing 2.5×RealMastermix (Tianwei, China), 10 μM of the gene-specific primer (F: 5′-GAAGCCCCCCAAATCTT-3′) and (R: 5′-CCTCACACGAGACAAGAAACAC-3′), 1/10 cDNA first-strand. Quantitative real-time PCR (qRT-PCR) was performed with an ABI PRISM^®^ 7000 system using the SYBR Green PCR master mix kit according to the manufacturer’s instructions. The relative amount of gene expression was calculated using the expression of β*-Tubulin* as internal standard. The following program was applied: initial template denaturation at 95°C for 2 min, followed by 40 cycles of denaturation at 95°C for 20 s, annealing at 60°C for 30 s and extension at 72°C for 40 s. Fluorescence signals were collected at each polymerization step. The specificity of the PCR amplification was checked with a melt curve analysis following the final cycle of the PCR.

The relative quantity of gene expression was detected using 2^-ΔΔC_t_^ method (Livak and Schmittgen, 2001).

### Development of *TaCRT-D*-overexpressing *Arabidopsis Plants*

The *TaCRT-D* coding sequence including *Sac*I (5′) and *Sal*I (3′) restriction sites was amplified using the forward primer 5′-TAGCGAGCTCACCACCACTTCCTCGTCTC-3′ and reverse primer 5′-TATCGTCGACTTCCCTCACACGAGACAAG-3′. The underlined sequences of the primers correspond to the *Sac*I and *Sal*I restriction sites, respectively. For the construction of the *TaCRT-D* overexpression vector, the entire cDNA sequence of *TaCRT-D* was PCR-amplified, then cleaved with *Sac*I and *Sal*I. The digested vector pCHF3 and the PCR fragment of *TaCRT-D* were ligated together to generate a new recombinant vector pCHF3-TaCRT-D. The binary vector was introduced into *Agrobacterium tumefaciens* strain GV3101 by electroporation (Gene Pulser II, Bio-Rad, Herlev, Denmark). The transformed agrobacteria were grown overnight at 28°C under spectinomycin (Spe) and rifampicin (Rif) selection. The positive clones were confirmed by restriction analysis, plasmid PCR, and sequencing. Individual clones were cultured in 100 ml of YEB liquid medium according to the ratio of 1:400 including 50 μg ml^-1^ spectinomycin (Spe) and 50 μg ml^-1^ rifampicin (Rif) at 28°C for 14 h with shaking (250 rpm). At OD_600_ ≈ 0.8 the bacteria were harvested by centrifugation (7000–7500 rpm, 15 min, 4°C). Cells were resuspended in one time the size of infiltration medium (5% Sucrose + 0.02% Silwet L-77). *Arabidopsis* plants were inverted and the flower buds were immersed into infiltration medium for 5 s. The transformed *Arabidopsis* plants were maintained under low light intensity and high humidity for 16–24 h, and then placed under normal light conditions until harvested. The positive transgenic plants were confirmed by detecting pCHF3-TaCRT-D using PCR and RT-PCR, and subsequently carried out phonotypical analyses.

### Examination of Seed Germination of *TaCRT-D* Transgenic *Arabidopsis* Lines

Seeds from different *TaCRT-D* transgenic *Arabidopsis* lines and the wild type lines were sowed evenly in the MS medium or MS medium supplemented with different concentration of NaCl, ABA and mannitol after disinfection, and then sealed with parafilm and placed in germination chamber. The number of germinated seeds and green cotyledons were recorded in 3, 6, 9, and 12 days. Three repeats were conducted for each treatment.

### Identification of Stress Tolerance of *TaCRT-D* Transgenic *Arabidopsis* Plants

To examine the function of *TaCRT-D* in plant stress responses, the *TaCRT*-*D*-overexpressing *Arabidopsis* lines and the wild type lines were cultured in MS solid medium for 10 days, then treated with 300 mM NaCl and 16.1% PEP, and the number of withered plants was counted. Leaf cell membrane stability index (MSI) (%) = (1-electrical conductivity before incubating/electrical conductivity after incubating)× 100.

In order to analyze the ABA effects on the transgenic seedling growth, we transferred seedlings cultured for 7 days to MS solid medium containing different concentrations of ABA (0, 10, and 15 μM), and observed the growth state of seedlings after 20 days.

### Primer Design and Cloning of *TaCRT-D* Genomic Sequence

Total genomic DNA was extracted from leaf samples. The genomic DNA primers (F: 5′-GTCTCCGGCGAGCCACC-3′, R: 5′-CACACGAGACAAGAAACACTTC-3′) for the full length amplification of the *TaCRT-D* gene were designed according to the full-length cDNA sequence cloned. Amplifications were performed first 3 min at 94°C followed by 32 cycles of: 1 min at 94°C, 1 min at 55°C and 4 min at 72°C. After the final cycle, the samples were incubated at 72°C for 5 min and hold at 4°C prior to analysis.

PCR products were excised from 1.2% agarose gels and purified with a PCR product purification kit DP-209-03 (Tiangen, Beijing). The purified products were then cloned using the pGEM-T Easy cloning vector (Tiangen, Beijing), and transformed into TOP10 competent *E. coli* cells by the heat shock method. Positive clones were selected by colony PCR and the plasmid DNA was extracted with plasmid extraction kit DP-103 (Tiangen, Beijing). DNA sequencing primers were a pair of universal primers (T7, M13R) and four stacked sets of primers (**Table 1**). DNA sequencing was performed in both directions by 3730XI DNA Analyzer (ABI) with the following program: initial denaturation at 96°C for 1 min; 34 cycles of 96°C for 10 s, 50°C for 5 s, 60°C for 3 min.

**Table 1:** Sequencing primers for *TaCRT* genes.

Primer	Sequence (5′→3′)	Loci (bp)
Flcrtseq1	GCAGGAAAATATTCTGGAGATC	807–829
Flcrtseq2	TGTGGGACTCAAACAAAGAAG	1503–1524
Flcrtseq3	AAGAAATAAAGTAGGATGAACGAC	2541–2565
Flcrtseq4	CTTGTCTTCTCTAGCTTTCCTCT	3152–3175

Based on the DNA variation of the *TaCRT* genomic sequences among the A, B and D genomes, three pairs of genome-specific primers were designed using the Primer Premier 5.0 software (**Table 2**). Primers AF/AR were used to amplify a 1473 bp fragment from the A genome, primers BF/BR to amplify a 913 bp fragment from the B genome, and primers DF/DR to amplify 633 bp and 715 bp DNA fragments from the A, B, D genome, respectively.

**Table 2:** Genome-specific primers used for chromosome assignment of the wheat *TaCRT* genes.

Primer	Sequence (5′→3′)	Chromosome	Expected	Ann.
		location	size (bp)	temp.(°C)
AF	GGTCATCTTCGAGGAGCGAT	3A	1473	55
AR	GCGTCAGCCTGTCAGTTTTA			
BF	GGTCATCTTCGAGGAGCGAT	3B	913	60
BR	TACTGGACCACCAATGTTTG			
DF	GTGGGACTCAAACAAAGAAG	3A 3B 3D	633 633 715	50
DR	TTAGAACTGAATGATGCATT			

The Chinese Spring nulli-tetrasomic lines, the common wheat and its wild relative species were used to determine the chromosome location of *TaCRT* genes. Genome-specific PCR was performed in a total volume of 15 μl containing 50 ng of genomic DNA, 2 × LA PCR buffer, 0.3 μM of each primer, 10 mM dNTP mix, and LA Tag Polymerase (5 U/μl). The PCR was carried out using PTC-100^TM^ Programmable Thermal Controller (MJ Research) as follows: initial denaturation at 94°C for 3 min; 32 cycles of 94°C for 1 min, an annealing step at variable annealing temperatures depending on the primer pairs for 1 min, 72°C for 2 min; and a final extension at 72°C for 10 min. The PCR products were electrophoresed on 2.5% agarose gels, stained with ethidium bromide and visualized under UV light.

### PCR-RFLP Marker Mapping

The primer pair F3/R6 (F3: 5′-TCGGTTCACAAAGGATGTC-3′, R6: 5′-TAATCGTGAATGGACATTCG-3′) were selected to amplify an upstream region (898 bp DNA fragment) of *TaCRT-D* gene, using Opata 85 and W7984 as template, respectively. Amplifications were performed first 3 min at 94°C followed by 32 cycles of: 1 min at 94°C, 1 min at 53°C and 4 min at 72°C. After the final cycle, samples were incubated at 72°C for 5 min and then hold at 4°C prior to analysis. The PCR products were purified and digested in the solution containing 1 μl 10× buffer, 10 U *Sal* I 0.25 μl, 4 μl DNA solution and 4.75 μl ddH_2_O. The genotypes of 114 RILs (Opata 85 × W7984) were assayed by PCR-RFLP with *Sal* I digestion. MAPMAKER/EXP version 3.0 was used to map *TaCRT*-*D* on chromosome.

## Results

### Expression Patterns of *TaCRT-D* in Response to Salt, ABA and Cold Stresses

**FIGURE 1:**
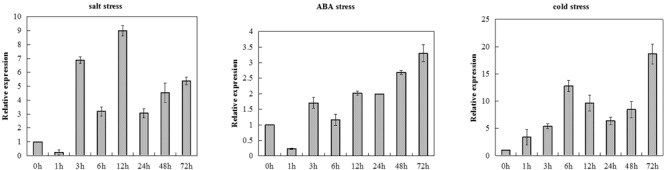
*TaCRT* expression levels in leaves sampled from common wheat seedlings under various stress treatments revealed by real-time RT-PCR analysis. The means were generated from three independent measurements and the bars indicated the standard errors.

The transcription levels of *TaCRT-D* were measured in salt, ABA and cold treated common wheat seedlings at different time points (**Figure 1**). *TaCRT-D* was induced by salt in the early stage of treatment. At 1 h after treatment, *TaCRT-D* mRNA levels were lower than the control. *TaCRT-D* mRNAs steadily increased from 3 h and reached the highest level at 12 h, which is 8.98 times that of the control. From 24 to 72 h, transcription amounts were lower than that of 12 h, but still higher than that of the control. *TaCRT-D* expression slightly increased in exogenous ABA treatment, and the transcripts was up to the peak at 72 h, showing 3.3 times as high as that in the control. *TaCRT-D* was up-regulated by cold stress in the early stage of treatment. During this process, *TaCRT-D* mRNAs steadily increased from 1 h and reached the first peak at 6 h with upregulation by eightfold compared to the control. Thereafter, the expression gradually decreased, down by 1/2 of the peak level at 24 h. And then *TaCRT-D* mRNA accumulation increase again, followed by the second peak at 72 h with 18.6-fold higher than that in the control.

### Seed Germination of *TaCR-D-*transgenic *Arabidopsis* under Different Stresses

**FIGURE 2:**
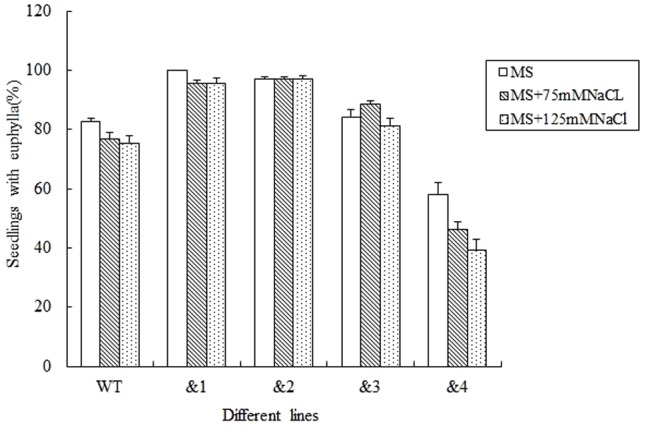
Germination and seedling growth of transgenic *TaCRT* lines and the wild type *Arabidopsis* exposed to NaCl. Euphylla (first pair of leaves) was counted from 12-day-old seedlings grown on MS agar plates supplemented with 0, 75, or 125 mM NaCl. The data represented the mean of the seed germination percentage from three independent experiments, in which 69 seeds were used for each line in each experiment.

For evaluating the capacity of transgenic lines resistant to stress, seed germination under different stresses was firstly identified. Wild-type lines and *TaCRT-D-*transgenic *Arabidopsis* lines were sowed in the MS medium with 0, 75 and 125 mM NaCl, respectively (**Figure 2**). The results showed that the seed germination percentage of the transgenic lines &1, &2 and &3 was higher than that of the wild type, while the germination percentage of the transgenic lines &4 was very low under different concentrations of NaCl stress. Seed germination numbers were statistically analyzed at 3, 6, 9, and 12 days after sowing, respectively. The results showed that there was no difference in germination rate between transgenic lines and wild-type seeds. 76.8% wild-type seed germinated after 12 days in MS medium supplemented with 75 mM NaCl, while 95.7% seeds of *TaCRT-D*-overexpression transgenic *Arabidopsis* line &1 germinated, 97.1% seeds of line &2 germinated, and 88.4 and 46.4% seeds of lines &3 and &4 germinated, respectively. When treated with 125 mM NaCl for 12 days, 75.4% wild-type seed germinated, while over 95% seeds of transgenic lines &1 and &2 germinated, 81.2% seeds of line &3 germinated, and only 39.1% seeds of line &4 germinated.

**FIGURE 3:**
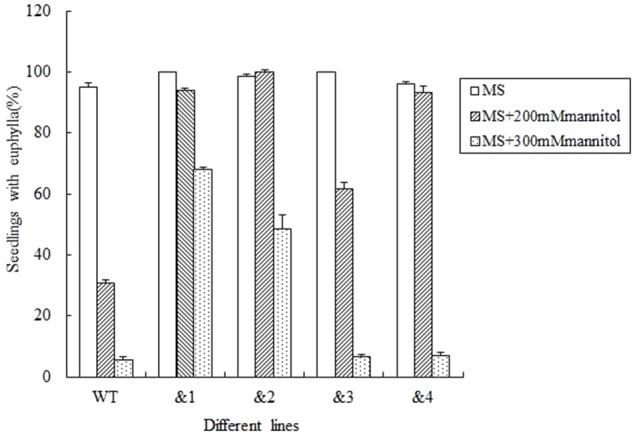
Seed germination and seedling growth of transgenic *TaCRT* lines and the wild type lines exposed to mannitol. Euphylla (first pair of leaves) were counted from 12-day-old seedlings grown on MS agar plates supplemented with 0, 200, or 300 mM Mannitol. The data represented the mean of the seed germination percentage from three independent experiments, in which 69 seeds were used for each line in each experiment.

To further determine if *TaCRT-D* was involved in the response to general osmotic effect stress, seeds were sowed in the MS medium with 0, 200 mM and 300 mM mannitol (an osmotic substance for simulating drought treatment) respectively, for 12 days. The growth of the control plants was greatly inhibited compared with the growth of transgenic plants, and it was more obvious when compared with the growth of transgenic lines &1 and &2 (**Figure 3**). The seed germination percentage of the transgenic lines &1 and &2 was 94.2 and 100%, respectively, much higher than that of the wild type (30.6%) in the MS medium with 200 mM mannitol.

**FIGURE 4:**
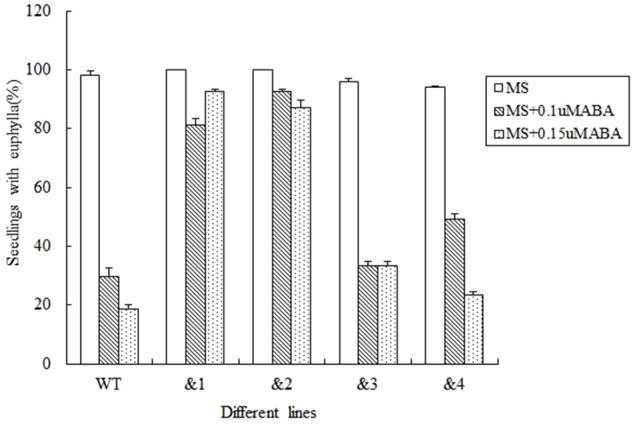
Seed germination and seedling growth of transgenic *TaCRT* lines and the wild type exposed to ABA. Euphylla (first pair of leaves) were counted from 12-day-old seedlings grown on MS agar plates supplemented with 0, 0.1, and 0.15 μM ABA. The data represented the mean of the seed germination percentage from three independent experiments, in which 69 seeds were used for each line in each experiment.

Abscisic acid plays an important role in regulating plant responses to various stresses (Finkelstein et al., 2002). Salt and drought responses in plants are triggered by the increased levels of ABA, which leads to the activation of a series of ABA-dependent responses (Zhu, 2002). In order to analyze the effect of ABA to the seed germination rate of the transgenic *Arabidopsis* lines, wild-type lines and *TaCRT-D-*transgenic *Arabidopsis* lines were sowed in the MS medium with 0, 0.1, and 0.15 μM, respectively. The results showed that after 12 days sowed in MS medium supplemented with 0.1 μM ABA, the seed germination percentage of the transgenic lines was higher than that of the wild type. After 0.15 μM ABA treatment for 12 days, the wild-type seed germination percentage was only 18.5%, but the seed germination of the transgenic lines &1 and &2 was about 90%, and the seed germination percentage of the transgenic lines &3 and &4 was 33.3 and 23.5%, respectively (**Figure 4**).

### Seedling Growth of *TaCRT-D-*transgenic *Arabidopsis* under Different Stresses

**FIGURE 5:**
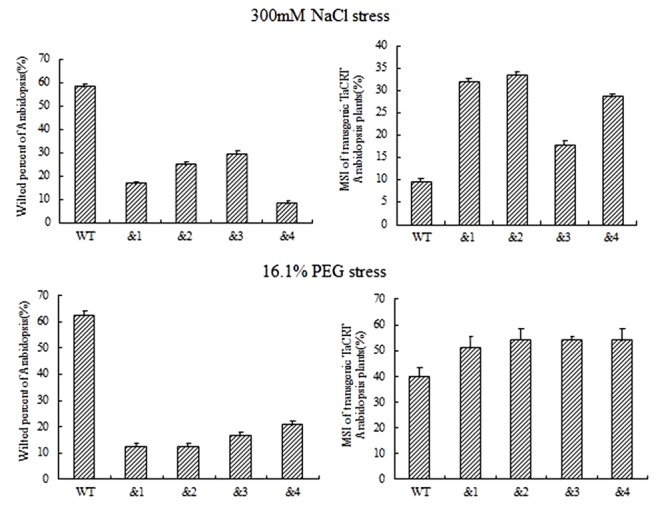
The growth of transgenic *TaCRT Arabidopsis* lines on the filter paper saturated with 300 mM NaCl or 16.1% PEG solution for 6 h.

To identify the cell membrane stability of *TaCRT*-*D*-overexpressing *Arabidopsis* under different stresses, 10-day-old seedlings were treated with NaCl (300 mM) and 16.1% PEG-6000 (-0.5 MPa) solution on filter paper. Symptoms of salt stress appeared on WT plants 6 h after treatments, but no sign of stress phenotype occurred on the transgenic plants. The percentage of the wilted leaves of the *TaCRT-D-*transgenic plants was <30%, whereas it was nearly 60% in WT plants. MSI (membrane stability index) of the transgenic lines was significantly higher than that of WT lines, strongly indicating that overexpression of *TaCRT-D* increased the cell membrane stability of *Arabidopsis* under NaCl and PEG stress (**Figure 5**).

**FIGURE 6:**
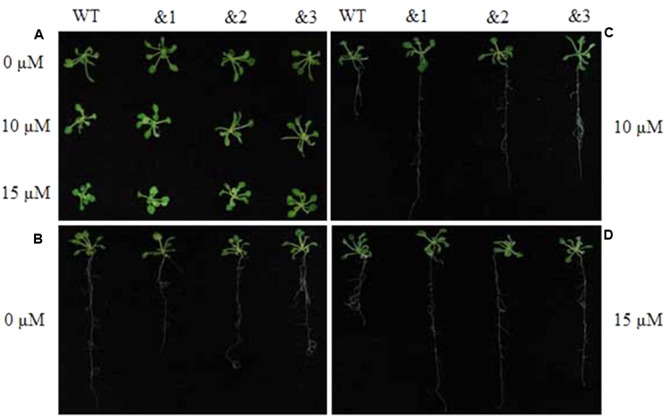
Enhanced resistance of the transgenic *TaCRT Arabidopsis* lines (&1, &2, &3) to abscisic acid (ABA). **(A)** The growth of wild type and transgenic *TaCRT* seedlings (&1, &2, &3) in treatment with different concentration of ABA for 20 days. **(B)** Root growth of wild type and transgenic *TaCRT Arabidopsis* seedlings (&1, &2, &3) on MS agar plates for 20 days. **(C)** Root growth of wild type and transgenic *TaCRT* seedlings (&1, &2, &3) sprayed 10 μM ABA for 20 days. **(D)** Root growth of wild type and transgenic *TaCRT* seedlings (&1, &2, &3) sprayed 15 μM ABA for 20 days.

To further evaluate the applicability of transgenic *TaCRT-D* seedlings to abiotic stress tolerance, the phenotypes were characterized under ABA stress. Wild-type lines and transgenic *TaCRT-D* lines were planted on MS medium for 7 days germination, and then carefully transferred to a new MS medium containing 0, 10 or 15 μM ABA. The results showed that the seedlings of WT *Arabidopsis* plants were slightly smaller than those of the transgenic plants after 20 days on MS medium containing 10 and 15 μM ABA (**Figure 6A**). The primary root lengths of the WT plants were longer than those of the transgenic lines on MS medium without ABA (**Figure 6B**). The primary root lengths of the transgenic lines were significantly longer than those of WT plants under 10 and 15 μM ABA treatments (**Figures 6C,D**).

### *TaCRT* Gene Isolation

**FIGURE 7:**
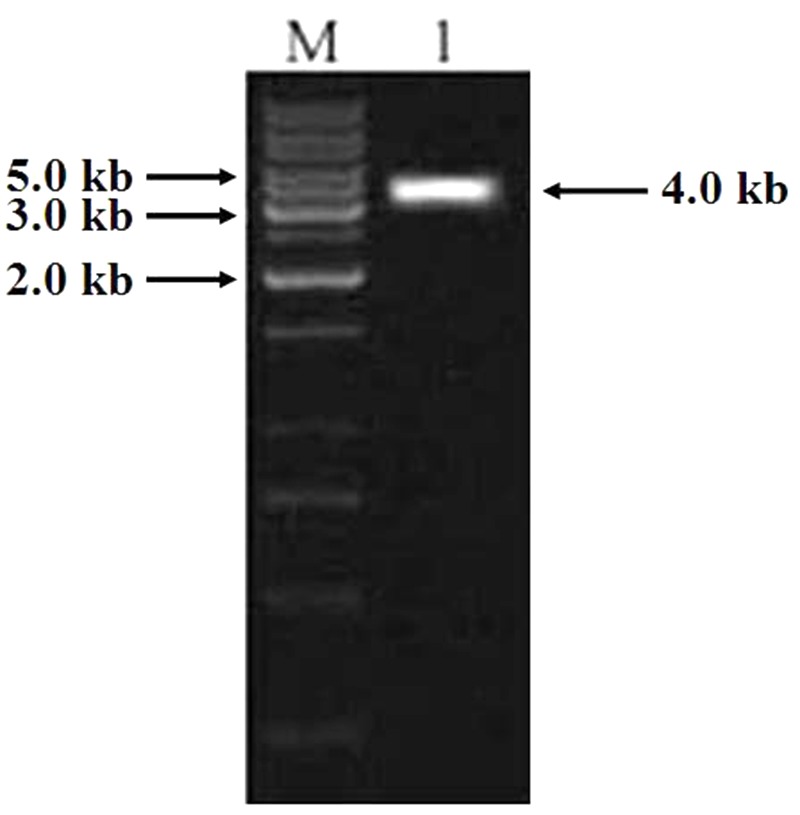
PCR product of full-length *TaCRT* gene. M: 1 kb marker; 1: PCR product of full-length *TaCRT* gene.

PCR was carried out using the genomic DNA isolated from “Hanxuan 10” as template. The 4000 bp band was purified and sequenced (**Figure 7**). The result showed that the band was composed of three sequences, namely *TaCRT-1, TaCRT-2*, and *TaCRT-3*, respectively, according to their sequence homologies with *Arabidopsis AtCRT1, AtCRT2*, and *AtCRT3*.

### *TaCRT* Sequence Polymorphism

The common wheat cultivar (cv.) Opata 85 (*Triticum aestivum* L., AABBDD, 2*n* = 6× = 42), synthetic wheat line W7984, the tetraploid *T. polonicum* cultivar PO9, the diploid accessions *T. urartu* U203 (AA, 2*n* = 2× = 14), *Aegilops speltoides* Y2003 (BB, 2*n* = 2× = 14), and *Aegilops tauschii* Y215 (DD, 2*n* = 2× = 14) were used for PCR amplification of *TaCRT* sequences. In addition, DNA sequences of the *TaCRT* gene from the corresponding genomes were also isolated. *TaCRT-1, TaCRT-2*, and *TaCRT-3* genomic sequences from the common hexaploid wheat were highly homologous with those of the diploid accessions *T. urartu* U203 (AA, 2*n* = 2× = 14), *Aegilops speltoides* Y2003 (BB, 2*n* = 2× = 14), and *Aegilops tauschii* Y215 (DD, 2*n* = 2× = 14). The similarities were 98.2, 99.7, and 99.5%, respectively. So it was deduced that the three sequences of *TaCRT* gene were from the A, B, and D genome of hexaploid wheat, namely *TaCRT-A, TaCRT-B*, and *TaCRT-D*, respectively.

**FIGURE 8:**
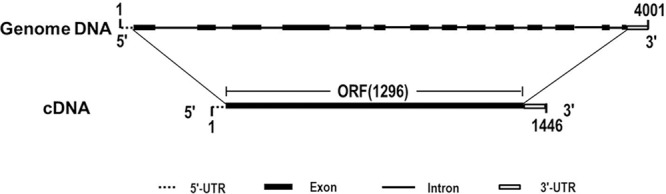
Genomic structure of *TaCRT-D* gene in wheat.

Due to the heterogeneous hexaploid in wheat genome, *TaCRT* gene exist multiple isoforms and DNA polymorphism in wheat A, B and D genome. The genome sequence variation was detected for different *TaCRT* isoforms. Here, we focused on *TaCRT-D* isoform (**Figure 8**) whose full-length genomic sequence was obtained from D genome in common wheat. The *TaCRT-D* full-length genomic sequence is 4001 bp, containing 14 exons, 13 introns, 5′-UTR (34 bp) and 3′-UTR (115 bp). The region of 14 exons at the DNA sequence was located at the following position, 35–161 bp, 733–841 bp, 979–1170 bp, 1481–1739 bp, 1914–1971 bp, 2058–2104 bp, 2430–2516 bp, 2601–2658 bp, 2765–2835 bp, 2947–3040 bp, 3119–3175 bp, 3290–3384 bp, 3754–3784 bp, and 3876–3886 bp, respectively.

**FIGURE 9:**
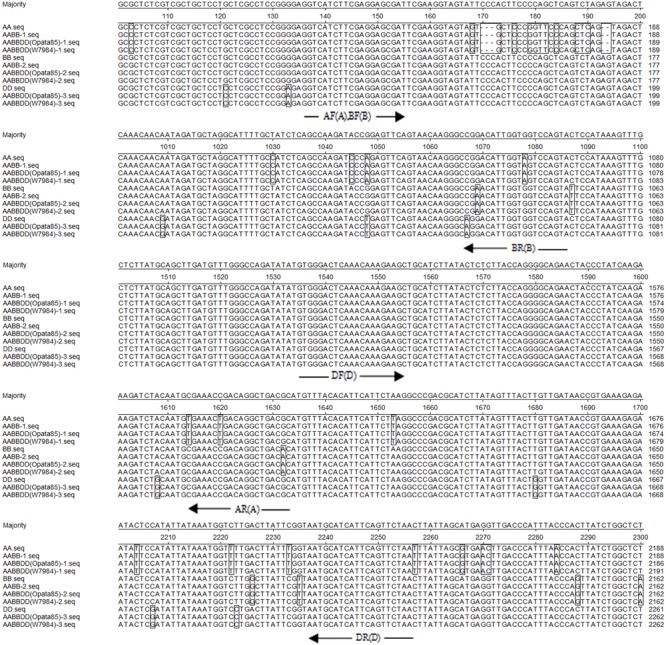
Partial alignment of the genomic sequences of *TaCRT* genes from different wheat accessions. AA, BB and DD are the *TaCRT* sequences from the diploid accessions: *T. urartu* U203, *Ae. speltoides* Y2003, and *Ae. tauschii* Y215; AABB-1 and AABB-2 are two *TaCRT* sequences from the tetraploid *T. polonicum* cultivar PO9; AABBDD-1, AABBDD-2 and AABBDD-3 are three *TaCRT* sequences from common wheat cv. Opata 85 and synthetic wheat W7984. The white background shows identity, boxes indicate non-homologies. The primer positions are indicated with arrows. The letters AF, AR, BF, BR, DF and DR represent the sequences for forward (F) and reverse (R) primers of A, B, and D genomes. Sequence segments with target loci are listed.

Nucleotide sequence alignment of the genomic sequences of *TaCRT* genes from hexaploid wheat (AABBDD) and its different accessions (AA, BB, DD, AABB) showed that there were length polymorphisms and single nucleotide polymorphisms (SNPs) among the *TaCRT-A, TaCRT-B*, and *TaCRT-D* loci (**Figure 9**).

### Development of Genome-Specific Primers and Determination of the Chromosome Location of *TaCRT-A, TaCRT-B* and *TaCRT-D* Homeologous Loci

**FIGURE 10:**
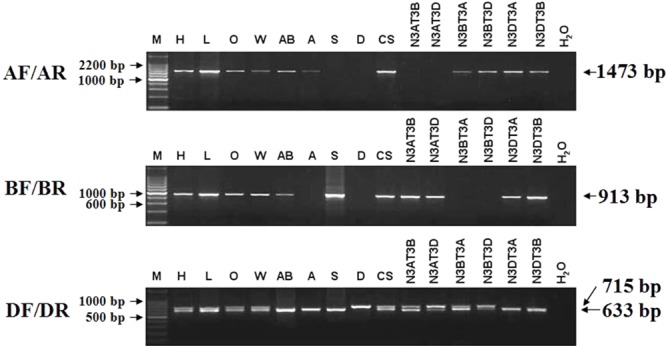
The location of *TaCRT* in the chromosome of wild relative species and nulli-tetrasomic lines of Chinese Spring. Absence of a band indicates the specific sequence is located on the corresponding null genome or chromosome. The size of the amplification product is shown on the right. H_2_O is a negative control, in which H_2_O is used as the template. For primer pair DF/DR, two PCR amplicons are identified in all hexaploid accessions, including Hanxuan 10, Lumai 14, Opata 85, W7984, Chinese Spring, and N3AT3B, N3AT3D and N3BT3A of Chinese Spring. However, only a smaller amplicon presents in *T. polonicum* cultivar PO9 (4×), *T. urartu* UR203 (2×), *Ae. speltoides* Y2003 (2×), N3DT3A and N3DT3B, which lack the D genome or chromosome 3D. The larger amplicon derived from the DF/DR primer pair is therefore 3D chromosome-specific.

Among the three *TaCRT* sequences in genome A, B, and D, there were abundant polymorphism sites between 1000 and 2300 bp. The genome-specific primers were designed based on the polymorphism in this region (**Figure 9**). The sequences of *TaCRT* homeologous loci in A, B, and D genomes were specifically amplified by the primer pairs from common wheat and its relatives. The AF/AR was designed to amplify a 1473 bp DNA fragment in the A genome (primer site is indicated in **Figure 10**). The BF/BR primers, which amplify a 913 bp DNA fragment, were designed as a B genome-specific primer pair. The DF/DR was selected to amplify sequences from all three genomes, with the amplifications of 633 bp DNA fragments in both A and B genomes and 715 bp in D genome (**Figure 10**).

To confirm these genome-specific primers, the genomic DNAs of Opata 85, synthetic W7984, *T. polonicum* cultivar PO9 and the three diploid accessions (*T. urartu* U203, *Ae. speltoides* Y2003, and *Ae. tauschii* Y215) were used as templates to amplify the proposed sequences. It confirmed that primer AF/AR was A genome-specific, BF/BR for B genome-specific, and DF/DR for D genome-specific with a longer amplification product (715 bp). The locations of the target genes in chromosomes were examined with different ploidy materials including the common hexaploid wheat “Hanxuan 10” and “Lumai 14,” and a set of nulli-tetrasomic lines developed in Chinese Spring. A further PCR-based analysis of nulli-tetrasomic lines also showed supportive evidence (**Figure 10**). Thus the primer sets AF/AR, BF/BR, and DF/DR were appropriate to be used as genomic functional makers for *TaCRT-A, TaCRT-B*, and *TaCRT-D* homeologous loci, respectively.

### Development of an Allele-Specific Functional Marker of *TaCRT-D* Isoform by PCR-RFLP

**FIGURE 11:**
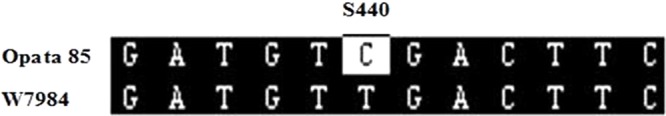
*Sal*I digestion site in the RIL population parents Opata 85 and W7984. To design the PCR-RFLP, *TaCTR* genes were amplified using the parents (Opata 85 and W7984) as templates, PCR products were digested with restriction enzymes *Sal*I, and then were analyzed using RFLP. Different bands appeared after both parents were digested (**Figure 12**). A 898 bp DNA fragment from D genome in Opata 85 by restriction endonuclease digestion and agarose gel, there were 2 *Sal*I restriction fragments, 593 and 305 bp, respectively.

**FIGURE 12:**
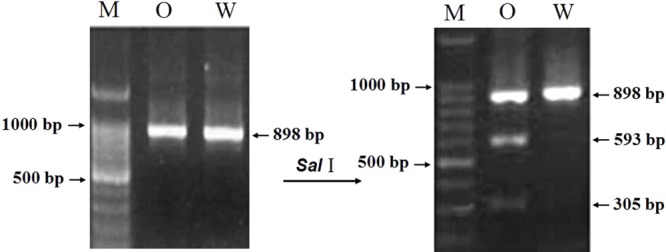
The result of restriction enzyme digesting the amplified PCR products between two parents of RIL population. M: DNA ladder 100; O: Opata 85; W: W7984.

For developing an allele-specific maker of *TaCRT-D* isoform on wheat chromosomes, sequence polymorphisms between the parents (Opata 85 and synthetic W7984) of the RILs were examined. Sequencing results showed that one SNPs, S440 (T→C), was detected in the D genome sequences of the parents (**Figure 11**), which was used to differentiate Opata 85 from W7984. More importantly, PCR product of *TaCRT-D* isoform from Opata 85 could be digested with restriction enzymes *Sal*I, whereas the product from W7984 could not be digested (**Figure 12**). Therefore, the primer sets used to detect this SNP and the corresponding *Sal*I-digested PCR products were developed as an allele-specific functional marker of *TaCRT-D* isoform.

### Fine Mapping *TaCRT-D* Locus

**FIGURE 13:**
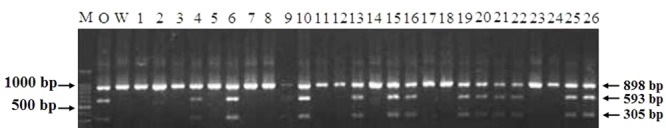
Genotypes of *TaCRT*-*D* in partial lines of RIL population using PCR-RFLP. M: DNA ladder 100; O: Opata 85; W: W7984; 1∼26: Partial lines of RIL population.

**FIGURE 14:**
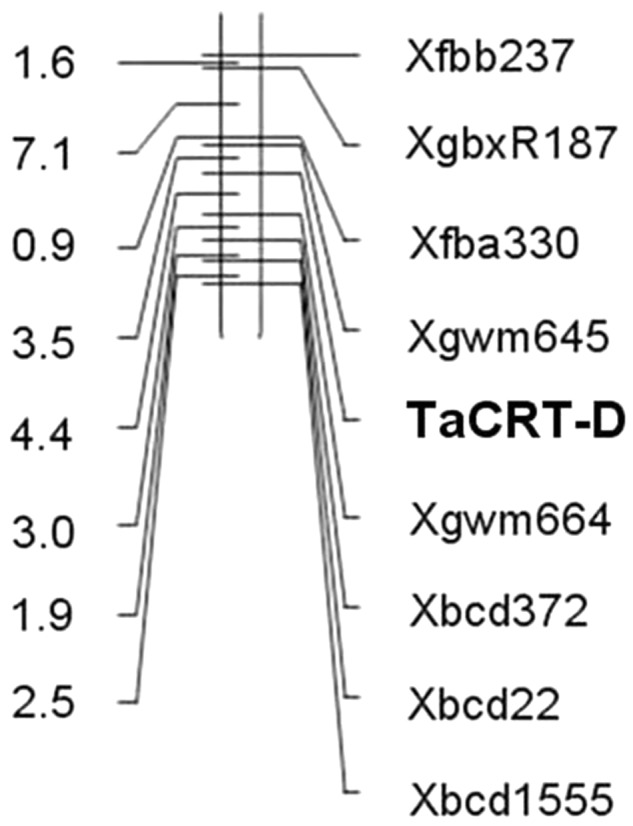
Map of *TaCRT-D* on chromosome 3DL of wheat.

The genotypes of 114 RILs developed from Opata 85 × W7984 were identified using restriction enzymes *Sal*I digestion of the PCR products. Apart from nine PCR reactions that could not detect the internal positive control, the genotypes of 52 lines were the same as that of W7984, and 53 lines showed the signal expected for *TaCRT-D* alleles in Opata 85 (**Figure 13**). Furthermore, the method was employed to fine map the *TaCRT-D* locus into the chromosome 3DL, where it was flanked by marker *Xgwm645* and *Xgwm664*, with genetic distances of 3.5 and 4.4 cM, respectively (**Figure 14**).

## Discussion and Conclusion

Wheat is sensitive to various abiotic stresses. Drought, cold, and salinity are the most important abiotic stress factors. They severely limit grain yield of wheat. This study was conducted to investigate wheat TaCRTs’ roles in stress tolerance. Another objective is to develop functional markers for *TaCRT* isoform so as to pyramid the excellent alleles in wheat breeding program.

### Overexpression of *TaCRT* Enhances Tolerance to Abiotic Stress in Wheat and *Arabidopsis*

Calreticulin is now recognized as a multifunctional and multicompartmental protein. Different isoforms of *CRT* may participate in different regulatory pathways and would function in different stress responses in plants (Jia et al., 2009). Persson et al. (2003) used *Arabidopsis* cell suspension cultures to obtain information about the regulation of the different CRT isoforms. *CRT3* showed a fast response to salt and tunicamycin, with a several-fold increase in expression for both treatments. In contrast, both *CRT1* and *CRT2* expression showed no major increase in response to 30-min treatments. Loss of all *AtCRT1, AtCRT2*, and *AtCRT3* reduced tolerance to water stress in *Arabidopsis* (Kim et al., 2013). Jia et al. (2008) found that *TaCRT3* was up-regulated expression in the PEG-stressed wheat seedlings and overexpression of *TaCRT3* increased tobacco plant drought tolerance. Xiang et al. (2015) found that wheat *CRT1/CRT2* and *CRT3* differed in their expression patterns during plant development and were all up-regulated by NaCl stress. In addition, the expression of *CRT* genes was up-regulated by other environmental stimuli such as cold (Borisjuk et al., 1998; Sharma et al., 2004) and pathogens (Qiu et al., 2012). In the present study, real-time RT-PCR analysis revealed that *TaCRT-D* gene was induced to express in wheat seedlings by high salinity (250 mmol L^-1^ NaCl), low temperature (4°C) stresses and abscisic acid (50 μmol L^-1^ ABA) treatment. Similar double-peaked expression patterns of *TaCRT-D* were identified in various stress responses (**Figure 1**). Significant differences in expression levels and response times indicate that *TaCRT-D* was highly sensitive to salt and cold stresses, and less sensitive to ABA treatment (**Figure 1**), suggesting that *TaCRT-D* may be involved in rapid response to NaCl and cold stresses.

To investigate further whether *TaCRT-D* is involved in other stress responses, we developed a number of transgenic *Arabidopsis* lines overexpressing the ORF of *TaCRT-D*. Seed germination and early seedling development were selected as the traits to assess the tolerance of the transgenic plants to abiotic stress. The results clearly demonstrated that *TaCRT-D* overexpression greatly increased seed germination percentage under different abiotic stresses including NaCl, mannitol and ABA (**Figures 2–4**). It should be noted that the germination rates of the four transgenic *Arabidopsis* lines were different under different stress treatments. Possible reason for this difference might be the effect of different insertion sites of the transgene in the host genome, because the expression of *TaCRT-D* gene was almost same in four transgenic lines. In view of this, we just selected transgenic lines &1 and &2 for subsequent analysis.

For early development of seedling, compared with the wild-type plants, *TaCRT-D*-overexpressing *Arabidopsis* grew much better, showing less wilt under NaCl and PEG stress (**Figure 5**). Such enhanced stress tolerance might result from the increase in cell membrane stability caused by *TaCRT-D* overexpression in *Arabidopsis* under abiotic stresses (**Figure 5**). In comparison to the WT control under stress conditions, the *TaCRT-D* transgenic seedling exhibited significantly higher MSI (**Figure 5**), a typical physiological parameters for evaluating abiotic stress tolerance and resistance in crop plants (Dhanda and Sethi, 1998; Farooq and Azam, 2006). In general, higher MSI in plants means stronger stress tolerance/resistance.

Phytohormones play crucial roles in plant growth and development as well as stress responses. It was founded that *CRT* gene expression was regulated by exogenous gibberellic acid (GA3) in barley aleuronic cells (Denecke et al., 1995). *CRT* expression was also detected to be involved in ABA-induced salt tolerance of potato (*Solanum tuberosum*) (Shaterian et al., 2005). In this study, *TaCRT-D* overexpression improved the development of *Arabidopsis* root system (**Figures 6C,D**) under ABA treatment, which subsequently could enhance the uptake of water and nutrients under osmotic stress. Taken together, our data evidence that TaCRT-D isoform functions importantly in multiple abiotic stress responses, unlike TaCRT1 mainly in salt stress (Xiang et al., 2015), and TaCRT3 in drought stress (Jia et al., 2008).

The known studies show that there are three subfamilies of Calreticulin (CRT) protein in *Arabidopsis thaliana*, namely AtCRT1, AtCRT2, and AtCRT3, respectively. Similarly, three subfamilies of CRT (TaCRT1, TaCRT2, and TaCRT3) was detected in wheat (*Triticum aestivum*) (Jia et al., 2008; An et al., 2011; Xiang et al., 2015). Due to heterologous hexaploid (AABBDD), each of TaCRTs has three homologous genes which are located in A, B, and D genomes, respectively. These TaCRTs can be named as TaCRT1-A, TaCRT1-B, TaCRT1-D, TaCRT2-A, TaCRT2-B, TaCRT2-D, TaCRT3-A, TaCRT3-B, and TaCRT3-D. In the present study, we cloned *TaCRT3* gene from D genome (namely *TaCRT-D*). More wheat *TaCRT* genes are needed to be cloned and characterized for further understanding TaCRTs functions in wheat biology.

### Functional Marker Development and Gene Mapping in Common Wheat

With the advent of DNA markers, marker-assisted selection has been developed as a strategy for accelerating the process of wheat breeding. Numerous conventional markers such as RFLP, RAPD, AFLP, and SSR have been identified in wheat, but most of them are not developed from the genes themselves because the wheat gene cloning is complicated by its allohexaploid (2*n* = 6× = 42) nature and large genome size. In contrast, functional markers (FMs) are usually designed from polymorphisms within transcriptional regions of functional genes. Such FMs are completely correlated with gene function (Andersen and Lübberstedt, 2003), irrespective of the complex chromosome structure. For example, Zhao et al. (2007) reported FMs for *LMW-GS* alleles at the *Glu-D3* and *Glu-B3* loci in bread wheat. FMs for polyphenol oxidase (PPO) genes on chromosomes 2A and 2D have been validated in common wheat (He et al., 2007). Therefore, FMs could dramatically facilitate the accurate selection of target genes in wheat breeding.

Single nucleotide polymorphism widely used in many research fields is superior to other markers based on the high density, widespread distribution, and straight-forward assay methods. Compared with diploid crops, development of gene-derived functional markers is more complex in wheat because of the allohexaploid consisting of the three high-homology genomes of A, B, and D. (Liu et al., 2012). It is difficult to use the difference between the homologous sequences to distinguish the different genomic distribution of the target gene in common wheat, and to perform the chromosomal localization and precise genetic mapping. In recent years, however, a number of SNP markers in wheat were developed, and further used for mapping and cloning of the related genes (Su et al., 2011; Zhang et al., 2014; Yue et al., 2015; Li et al., 2016). Sequence polymorphism of the target gene among different genomes in wheat was usually used to develop the genome-specific primers. For example, the genome-specific primers for a series of starch biosynthesis genes including *Agp-L, SUT, Wx, Agp-S* and *SsI* were developed by this way (Blake et al., 2004). Similarly, Zhao et al. (2004) designed genome-specific PCR primer pairs for *LMW-GS* gene based on alignment of eleven *LMW-GS* gene sequences in the EMBL and the GenBank, and ultimately cloned *LMW-GS* genes at *Glu-D3* loci. Wei et al. (2009) designed five primer pairs for *Dreb-B1* gene and mapped this locus on wheat chromosome 3BL. Wang et al. (2011) mapped the *TaPP2Aa* gene on chromosome 5B and 5D in wheat.

Here, we designed the genomic-specific primers based on the polymorphism of *TaCRT* genes. The PCR with these genome-specific primers was employed to clone the *TaCRT* homologous gene sequences (*TaCRT-A, TaCRT-B*, and *TaCRT-D*) in all three genomes A, B, and D. The three *TaCRT* genes were finally located to chromosomes 3A, 3B, and 3D using nulli-tetrasomic lines. For example, the *TaCRT-A* was mapped between SSR markers *Xmwg*30 and *Xmwg*570 on chromosome 3A. *TaCRT-D* was mapped to chromosome 3DL between markers *Xgwm645* and *Xgwm664* using a PCR-RFLP functional marker based on the SNPs in this locus. To our best knowledge, it is the first time to develop genome-specific and allele-specific functional makers for wheat *TaCRT* genes, which could be used for marker-assistant selection in wheat breeding program.

## Conclusion

The *TaCRT-D* transcripts were induced to express by high salinity, low temperature and ABA stress in wheat seedlings. Over-expression of *TaCRT-D* in heterologous *Arabidopsis* plants enhanced the transgenic plant abiotic stress resistance at seed germination stage and seedling stage under various environmental stresses. These results indicate that *TaCRT-D* plays an important role in wheat responses to abiotic stresses. Furthermore, genome-specific and allele specific markers were developed for cloning and mapping of *TaCRT-A, TaCRT-B* and *TaCRT-D* genes in common hexaploid wheat. Notably, *TaCRT-D* was mapped between *Xgwm645* and *Xgwm664* on the 3DL with 3.5 and 4.4 cM distance from the marker. Such data will expand our knowledge about wheat CRTs’ functions, providing the target gene and the related functional markers for gene modification and marker-assistant breeding to increase wheat stress tolerance.

## Author Contributions

RL and RJ conceived and designed the experiments. JW performed the gene functional marker mapping and the gene functional analysis. XM analyzed the gene mapping data. RJ and JW provided project resources. JW and RJ wrote the manuscript.

## Conflict of Interest Statement

The authors declare that the research was conducted in the absence of any commercial or financial relationships that could be construed as a potential conflict of interest.
